# Clear Aligners and Bruxism: A Systematic Review

**DOI:** 10.1111/joor.70189

**Published:** 2026-03-17

**Authors:** André Luís Porporatti, Ângela Graciela Deliga Schroder, Milena Sampaio Kuczera, Aline Bastos de Barros, Yves Boucher

**Affiliations:** ^1^ Laboratory “Oral Health” UMR 1333 Inserm Faculté de Santé UFR Odontologie Université Paris Cité Montrouge France; ^2^ NARSM (Núcleo de Revisão Sistemática e Meta‐análise) – Tuiuti University Curitiba Brazil; ^3^ Orthodontic Clinical Practice Lisbon Portugal; ^4^ Service Odontologie Hôpital Pitié‐Salpêtrière (AP‐HP) Paris France

**Keywords:** aligners, association, bruxism, systematic review

## Abstract

**Background:**

Bruxism is a masticatory muscle activity that may occur during sleep (SB) or wakefulness (AB) and is not classified as a movement or sleep disorder. Clear aligner therapy, widely adopted as a removable and aesthetic orthodontic alternative, modifies occlusal contacts and mandibular dynamics, which may influence the occurrence or perception of bruxism behaviours.

**Objective:**

To systematically review the association between clear aligner therapy and bruxism (SB, AW and mixed/self‐reported), with a focus on evaluating its role as neutral, protective or a potential risk factor.

**Methods:**

A systematic search of electronic databases and grey literature was conducted through April 2025, with included studies that identified bruxism using validated instrumental methods (portable electromyography for SB, or smartphone‐based ecological momentary assessment for AB), subjective questionnaires on behaviours and related symptoms. Risk of bias was assessed using the Joanna Briggs Institute tool, and evidence quality was evaluated with GRADE.

**Results:**

Eleven studies evaluated 818 patients (72.8% female). Most studies suggested a neutral effect of clear aligners on bruxism. For SB, aligners often reduced tonic contractions but showed inconsistent effects on higher phasic activity and no changes on overall SB index. AB was generally unaffected, though one study reported reduced self‐reported parafunctions with altered muscle recruitment. Mixed/self‐reported bruxism outcomes were conflicting: some studies indicated improvements in clenching and TMJ pain, while others found a higher prevalence of bruxism symptoms during aligner therapy. Methodological heterogeneity and risk of bias were notable, precluding that no meta‐analysis was possible. GRADE certainty for randomised studies was moderate and very low for non‐randomised studies.

**Conclusion:**

Current evidence on the relationship between clear aligner therapy and bruxism remains limited and heterogeneous. Most studies suggest a neutral effect, with occasional reductions in tonic contractions or symptom improvement, but also reports of increased phasic activity or self‐reported bruxism. Overall, aligners cannot yet be classified as protective or harmful, highlighting the need for standardised, high‐quality studies to clarify their role in bruxism management.

## Graphical abstract

This study aimed to systematically evaluate the relationship between clear aligner therapy and bruxism, including both sleep and awake bruxism, to determine whether aligners act as a neutral factor, protective agent, or risk factor. The findings indicate that no definitive conclusion can be drawn, as the current evidence is limited and contradictory. Overall, there is still no solid scientific consensus regarding the impact of clear aligners on bruxism behaviour.

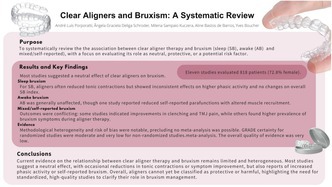

## 1 Introduction

Bruxism is a masticatory muscle activity that is characterised as rhythmic (phasic) or non‐rhythmic (tonic) when in sleep (SB); and by repetitive or sustained tooth contact and/or by bracing or thrusting of the mandible when during wakefulness (AB); and is not a movement disorder or a sleep disorder [1]. Both conditions may contribute to tooth wear, restorative failure, temporomandibular disorders (TMD) and orofacial pain, representing a relevant challenge in dental practice [2].

The advent of clear aligner therapy has revolutionised orthodontic treatment by providing a removable, aesthetic and patient‐friendly alternative to conventional fixed appliances [3]. Beyond their orthodontic purpose, aligners alter occlusal contacts and mandibular dynamics, potentially influencing the occurrence or perception of bruxism behaviours [4]. Unlike stabilisation splints, which are designed to protect the dentition and sometimes modulate muscle activity [5], aligners are thinner, usually soft, cover both arches and are worn continuously during treatment. This has led to speculation that aligners could either mitigate, exacerbate or have no significant effect on bruxism.

Several studies have investigated the interaction between clear aligners and bruxism‐related outcomes. Evidence, however, remains inconsistent. Some trials suggest that aligners may reduce tonic episodes of SB or modify muscle activity patterns [6] whereas others indicate no significant changes in AB frequency [7]. In addition, reports on patient‐reported symptoms such as muscle pain, temporomandibular joint discomfort and occlusal sensitivity vary widely across populations and methodologies [8, 9, 10].

The heterogeneity of study designs, outcome measures and follow‐up periods poses a challenge for clinicians seeking evidence‐based guidance on whether aligners can be considered neutral, protective, or risk factors in patients with bruxism. Moreover, the overlap between orthodontic effects, placebo expectations and natural variability of bruxism makes interpretation even more complex. A systematic appraisal of the literature is therefore needed to clarify current evidence and identify research gaps. Therefore, the aim of this study was to systematically review the association between clear aligner therapy and bruxism, with a focus on evaluating its role as neutral, protective, or a potential risk factor.

## 2 Methods

### 2.1 Protocol and Registration

This systematic review conformed to the Preferred Reporting Items for Systematic Reviews and Meta‐Analyses PRISMA Checklist [11]. The protocol was registered in the international Prospective Register of Systematic Reviews (PROSPERO) under number CRD420251053793.

### 2.2 Eligibility Criteria

We included studies that evaluated the association between the use of aligners and the presence of bruxism. In accordance with contemporary consensus definitions [1], studies were considered eligible when they evaluated the presence or modification of SB or AB using validated instrumental, behavioural, or self‐reported approaches. In addition, we also included investigations that assessed masticatory muscle activity through recording masticatory muscle activity or portable EMG‐based devices. We included studies that detected the presence of bruxism based on three main approaches. First, validated instrumental methods, including portable EMG combined or not with electrocardiography (ECG) for the assessment of SB and ecological momentary assessment through a smartphone application for AB. Second, subjective questionnaires, in which participants reported the frequency of bruxism behaviours and related symptoms such as muscle or temporomandibular joint pain. Finally, recording masticatory muscle EMG used during standardised tasks, providing additional insights into muscular recruitment patterns associated with parafunctional behaviours. Overall, the inclusion criteria were based on the PECOS methodology [12]: Participants (P) included children and adults undergoing orthodontic treatment; the Exposure (E) was the use of clear aligners; the Comparisons (C) were fixed orthodontics or no comparison group; the Outcomes (O) were the presence or modification of SB or AB, as assessed by instrumental, clinical, or self‐reported measures; and the eligible Study designs (S) were randomised clinical trials, observational studies (case–control, cross‐sectional and cohort), and case series with more than 10 cases.

The exclusion criteria encompassed: (1) Studies with no use of aligners; (2) Studies in animals; (3) Literature reviews, abstracts from conferences, letters, case reports (< 10 cases) and personal opinions; (4) full‐text not retrieved.

### 2.3 Information Sources and Search

Detailed individual search strategies were developed, in English, for each bibliographic electronic database: EMBASE, PubMed (Including Medline), Scopus and Web of Science. A grey literature search was performed on Google Scholar and Open Grey. All database searches were conducted from the starting coverage date through April 15th, 2025. More information on the search strategies was provided in Appendix S1 (which can be found online). Furthermore, the authors hand‐searched the reference lists of the selected articles for any additional references that might have been missed in the database searches. All references were managed, and the duplicated hits were removed by reference manager software (EndNote X7 Basic‐Thomson Reuters, New York, USA).

### 2.4 Study Selection and Data Collection Process

Study selection followed a two‐phase process. In phase one, two authors (A.L.P. and A.G.D.S) independently evaluated the titles and abstracts of all identified electronic database citations. In phase two, the same authors evaluated full‐text data. They independently screened papers in phases one and two, applied the eligibility criteria, collected key information from the selected studies, and cross‐checked the information. The selection from phase two was based solely on the full‐text assessment of the studies. When disagreement appeared, a third author (Y.B.) was involved to make a final decision about the inclusion or exclusion of studies.

For each of the included studies, the following items were recorded: author(s), year of publication, country, study design, characteristics of the study, characteristics of groups, criteria for evaluating and diagnosing bruxism, type of aligners used, main results and conclusions of the work. When the required data were not complete, the reviewers (A.L.P. and A.G.D.S) attempted to contact the study authors to retrieve any unpublished information. Three attempts were made in a 30‐day period, by email for the first, second and last author.

### 2.5 Risk of Bias in Individual Studies

The methodological quality of the studies was assessed using the Joanna Briggs Institute (JBI) Critical Appraisal Tools for cross‐sectional, cohort and case–control studies. Two reviewers (A.L.P. and A.G.D.S.) independently evaluated the risk of bias, rating each item as ‘yes’, ‘no’, ‘unclear’, or ‘not applicable’. Discrepancies were resolved by a third reviewer (Y.B.). Studies were categorised as having a ‘high’, ‘moderate’, or ‘low’ risk of bias based on the proportion of ‘yes’ responses. Specifically, 0%–49% indicated high risk, 50%–69% indicated unclear risk and ≥ 70% indicated low risk.

### 2.6 Study Outcomes and Summary Measures

Bruxism outcomes could be evaluated using both objective and subjective methods. For SB, studies could employ instrumental approaches such as the Bruxoff system, [13] which quantified the SB index and differentiated tonic and phasic contractions, as well as portable EMG to measure amplitude, frequency and duration of contractions [14]. AB could be assessed through ecological momentary assessment using smartphone‐based applications to record event frequency in real time or by combining EMG [15]. In addition, some studies could rely on self‐reported questionnaires, such as the Oral Behavioural Checklist (OBC) focusing on the perceived presence and prevalence of bruxism behaviours and related symptoms, including muscle pain, temporomandibular discomfort and headache [14, 16].

### 2.7 Synthesis of Results

Statistical pooling of data using meta‐analysis was planned whenever trials were considered combinable and relatively homogeneous in relation to design, interventions and outcomes. Heterogeneity between studies was evaluated either by considering clinical (differences about populations and results), methodological (design and risk of bias) and statistical characteristics (effect of studies) or by using inconsistency indexes (*I* ^2^) [17].

#### 2.7.1 Classification of Study Outcomes

For qualitative synthesis, studies were categorised by the authors according to the direction of their main bruxism‐related outcomes as neutral, protective or risk in relation to clear aligner therapy. This classification was defined a priori and applied consistently across all included studies. A study was considered neutral when aligner use did not result in statistically significant changes in bruxism frequency, SB index, AB behaviours or related EMG parameters compared with baseline or control conditions. A protective classification was assigned when aligner therapy was associated with a statistically significant reduction in at least one core bruxism‐related outcome, such as tonic contractions, clenching frequency, or validated questionnaire scores, regardless of whether this effect was transient. A study was classified as risk when aligner use was associated with a statistically significant increase in bruxism frequency, phasic muscle activity or self‐reported bruxism prevalence. This categorisation was intended solely as a structured qualitative framework to summarise heterogeneous findings and does not imply long‐term clinical benefit or harm. Given the variability in study design, diagnostic methods and follow‐up duration, the classification should be interpreted with caution and in conjunction with the narrative synthesis.

### 2.8 Confidence in Cumulative Evidence

A summary of the overall certainty of evidence available was presented using ‘Grading of Recommendations Assessment, Development and Evaluation’ (GRADE) Summary of Findings (SoF) tables, using GRADEpro software [17].

## 3 Results

### 3.1 Study Selection

**FIGURE 1:**
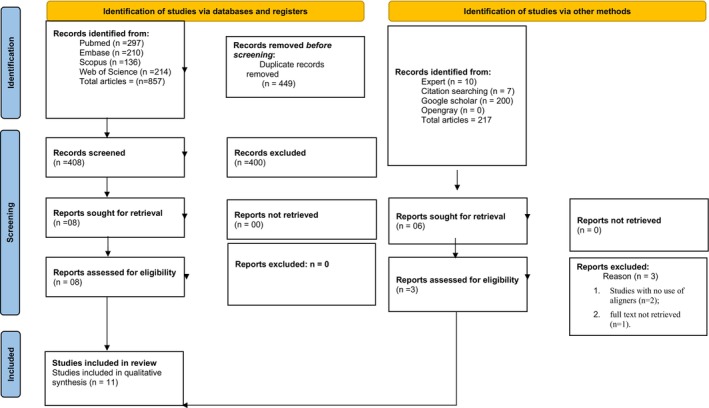
PRISMA 2020 flow diagram for new systematic reviews which included searches of databases, registers and other sources. From: Page et al. [18]. For more information, visit: http://www.prisma‐statement.org/.

The initial database search identified 857 studies. After removing duplicates, 408 studies remained; 400 of them were excluded after title and abstract revision, resulting in a final number of eight articles. Furthermore, 200 studies were found with Google Scholar, and none with OpenGrey. Seven studies were selected from a search of the reference lists of the selected studies, and ten studies were considered based on experts' suggestions. Furthermore, six of them were selected for full‐text review. Consequently, 14 studies became part of phase 2. During this phase, 03 studies were excluded (reasons for exclusion may be found in Appendix S2). Eleven studies were included for qualitative synthesis. A flowchart of the process of identification, inclusion and exclusion of studies is shown in Figure 1.

### 3.2 Study Characteristics and Results of Individual Studies

Across the 11 studies, a total of 818 patients were evaluated. Information on sex distribution was available for 552 participants, of whom 402 were female, corresponding to 72.8%. The proportion of women varied widely across studies, ranging from 39.5% in Pereira et al. [19] to 100% in Liu et al. [20], with intermediate values such as 47.2% in Bargellini et al. [21], 58.8% in Bargellini et al. [6], 62.5% in Colonna et al. [7], 58.0% in Heleiwa‐Ferioli et al. [22], 73.7% in Manfredini et al. [23] and 91.9% in Saccomanno et al. [9].

Included studies were conducted in five countries. Most of them were performed in Italy [6, 7, 8, 9, 10, 21, 23], while single studies were carried out in Spain [22], China [20], Brazil [19], and a New Zealand–based research team [24].

Regarding study design, five were randomised clinical trials [7, 10, 19, 20, 23], and six were non‐randomised: two were cohort studies [6, 24], two were cross‐sectional studies [8, 9], one was a case–control study [21], and one was a quasi‐experimental study [22]. All studies were published in English.

Five studies investigated SB using instrumental approaches such as portable EMG combined with electrocardiography (Bruxoff) or home‐based EMG devices [6, 10, 21, 23, 24]. Three studies focused on AB, employing ecological momentary assessment through smartphone applications or EMG combined with behavioural checklists [7, 19, 20]. The remaining three studies relied on self‐reported questionnaires that did not differentiate between SB and AB, but rather assessed the perceived presence of bruxism behaviours and related symptoms such as muscle pain, temporomandibular joint discomfort, or headache [8, 9, 22].

Most studies employed Invisalign aligners planned with ClinCheck software, either in standard or SmartTrack material [6, 8, 10, 19, 20, 21, 22]. Other brands included F22 aligners [7] and Zendura FLX passive clear aligners [24], while some studies referred only to clear aligners without specifying the manufacturer [9]. In addition, Manfredini et al. [23] investigated invisible orthodontic retainers rather than active aligner therapy.

#### 3.2.1 Results From Randomised Studies

Most studies found that clear aligner therapy does not significantly alter bruxism frequency or EMG indices when compared to fixed appliances or placebo splints. Pereira et al. [19] reported no change in AB frequency during 6 months of treatment. Colonna et al. [7] confirmed that neither passive nor active aligners influenced AB behaviours in the short term. Similarly, Manfredini et al. [23] observed no significant difference in SB indices or masseter activity with or without passive retainers made of aligner‐like material. Castroflorio et al. [10] found that full‐coverage aligners modified EMG contraction patterns, slightly increasing phasic and tonic episodes, but without changing SB frequency. Only Liu et al. [20] reported a reduction in oral behavioural checklist scores after 3 and 6 months of aligner use, suggesting a temporary improvement in parafunctional awareness.

#### 3.2.2 Results From Non‐Randomised Studies

Bargellini et al. (2017, case–control) showed a transient reduction in SB episodes after 1 month of aligner therapy, returning to baseline by 3 months. In a cohort follow‐up, Bargellini et al. [6] found that clear aligners did not change SB indices but reduced tonic contractions after 6 and 12 months. Pittar et al. [24] observed a short‐term decrease in EMG episode amplitude without changes in contraction frequency, suggesting a mild neuromuscular adaptation. Cross‐sectional studies [8, 9] revealed that self‐reported bruxism and muscle discomfort were frequent during aligner therapy, affecting about half of patients, though pain intensity was generally low and transient. The quasi‐experimental study by Heleiwa‐Ferioli et al. [22] reported a subjective decrease in clenching and masticatory muscle tension during Invisalign treatment, particularly in adults aged 28–36 years.

### 3.3 Study‐Specific Risk of Bias

**FIGURE 2:**
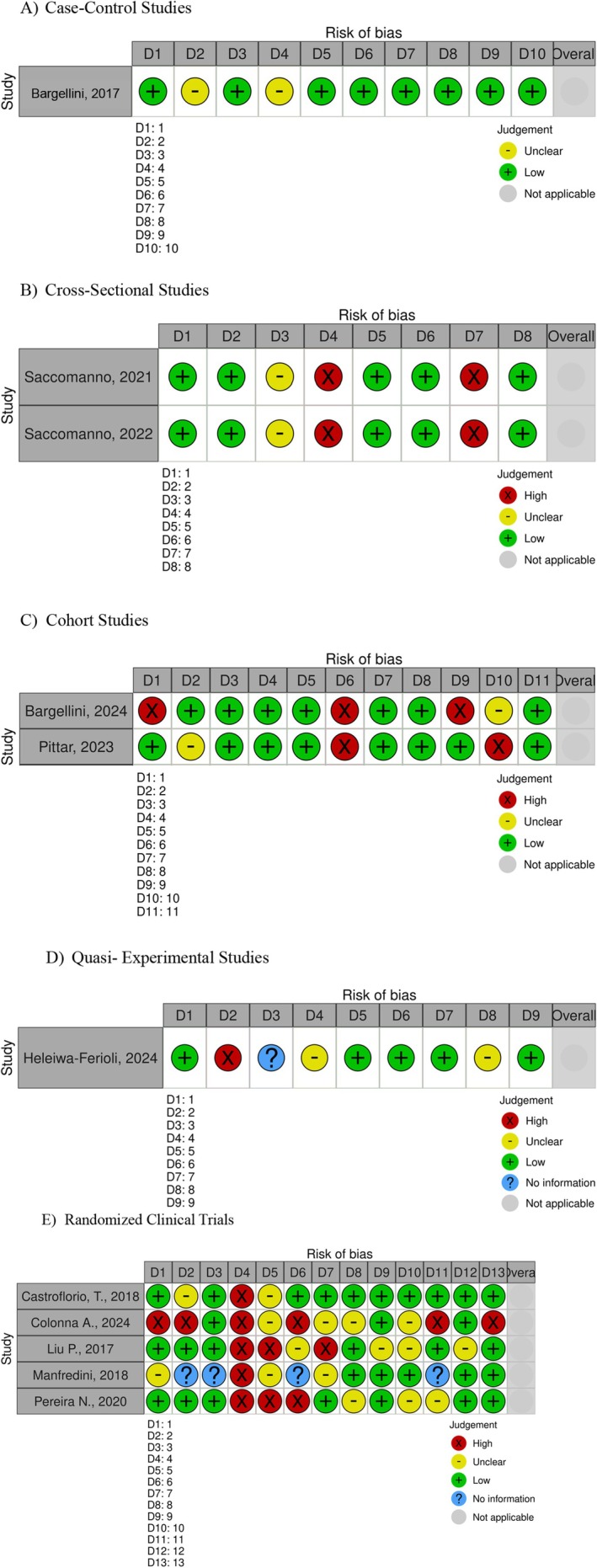
Critical appraisal tool to assess risk of bias summary. (A) Case–control studies. (1) Were the groups comparable other than the presence of disease in cases or the absence of disease in controls? (2) Were cases and controls matched appropriately? (3) Were the same criteria used for identification of cases and controls? (4) Was exposure measured in a standard, valid and reliable way? (5) Was exposure measured in the same way for cases and controls? (6) Were confounding factors identified? (7) Were strategies to deal with confounding factors stated? (8) Were outcomes assessed in a standard, valid and reliable way for cases and controls? (9) Was the exposure period of interest long enough to be meaningful? (10) Was appropriate statistical analysis used? (B) Cross‐sectional studies. (1) Were the criteria for inclusion in the sample clearly defined? (2) Were the study subjects and the setting described in detail? (3) Was the exposure measured in a valid and reliable way? (4) Were objective, standard criteria used for measurement of the condition? (5) Were confounding factors identified? (6) Were strategies to deal with confounding factors stated? (7) Were the outcomes measured in a valid and reliable way? (8) Was appropriate statistical analysis used? (C) Cohort studies. (1) Were the two groups similar and recruited from the same population? (2) Were the exposures measured similarly to assign people to both exposed and unexposed groups? (3) Was the exposure measured in a valid and reliable way? (4) Were confounding factors identified? (5) Were strategies to deal with confounding factors stated? (6) Were the groups or participants free of the outcome at the start of the study or at the moment of exposure? (7) Were the outcomes measured in a valid and reliable way? (8) Was the follow up time reported and sufficient to be long enough for outcomes to occur? (9) Was follow up complete, and if not, were the reasons for loss to follow up described and explored? (10) Were strategies to address incomplete follow up utilised? (11) Was appropriate statistical analysis used? (D) Quasi‐experimental studies. (1) Is it clear in the study what is the cause and what is the effect, that is, there is no confusion about which variable comes first? (2) Was there a control group? (3) Were participants included in any comparisons similar? (4) Were the participants included in any comparisons receiving similar treatment or care, other than the exposure or intervention of interest? (5) Were there multiple measurements of the outcome, both pre and post the intervention or exposure? (6) Were the outcomes of participants included in any comparisons measured in the same way? (7) Were outcomes measured in a reliable way? (8) Was follow up complete and if not, were differences between groups in terms of their follow up adequately described and analysed? (9) Was appropriate statistical analysis used? (E) Randomised clinical trials. (1) Was true randomisation used for assignment of participants to treatment groups? (2) Was allocation to treatment groups concealed? (3) Were treatment groups similar at the baseline? (4) Were participants blind to treatment assignment? (5) Were those delivering the treatment blind to treatment assignment? (6) Were treatment groups treated identically other than the intervention of interest? (7) Were outcome assessors blind to treatment assignment? (8) Were outcomes measured in the same way for treatment groups? (9) Were outcomes measured in a reliable way? (10) Was follow up complete and if not, were differences between groups in terms of their follow up adequately described and analysed? (11) Were participants analysed in the groups to which they were randomised? (12) Was appropriate statistical analysis used? (13) Was the trial design appropriate and any deviations from the standard RCT design, such as individual randomisation and parallel groups, accounted for in the conduct and analysis of the trial?

The risk of bias varied across the included studies. Based on the JBI Critical Appraisal Tools, one study was classified as moderate, and ten as high risk. Case–control studies were judged to have a moderate risk of bias, mainly due to limitations in controlling for confounding factors and incomplete adjustment for baseline characteristics. Cross‐sectional, cohort and quasi‐experimental studies were classified as having a high risk of bias, largely attributable to their observational nature, reliance on self‐reported outcomes, lack of temporal sequencing and limited control of confounders. Randomised clinical trials also showed a high risk of bias, primarily related to small sample sizes, short follow‐up periods, and incomplete reporting of allocation concealment and blinding procedures. A detailed breakdown of all appraisal items is presented in Figure 2.

### 3.4 Results of Individual Studies

Table 1 presents the main findings and conclusions of the studies.

**TABLE 1:** Summary of descriptive characteristics of included articles (*n* = 11).

First author, publication date, journal, country, study design	Characteristics of the study	Characteristics of groups	Criteria for evaluating and diagnosing bruxism	Type of aligners used	Main results	Conclusions of the work
Bargellini (2017) *Iranian Journal of Orthodontics* Italy Case–control	All the subjects were screened for sleep bruxism with a validated portable device (Bruxoff, OT Bioelettronica, Torino, Italy) (20–24). On the basis of this evaluation the subjects were divided into two groups: the case group composed by 20 patients (7 m, 13 f, 25 ± 10 years), and the control group composed by 33 patients (20 m, 13 f, 26 ± 10 years). The control group was a ‘waiting list’ control group, without any form of treatment for the following 6 months. During the follow‐up period, 13 patients from the control group (5 m, 8 f, 28 ± 7 years) withdraw from the study, obtaining a final control group formed by 20 patients (15 m, 5 f, 29 ± 12 years). Then other recordings were made at screening time, at the beginning of orthodontic treatment (only for case group) (T0), after 1 month (T1) and after 3 months (T2)	53 subjects (27 m, 25 f, 28 ± 13 years) Aligner group 20 patients (7 m, 13 f, 25 ± 10 years) Control group 33 (20 m, 13 f, 26 ± 10 years)	Portable device (Bruxoff, OT Bioelettronica, Torino, Italy) Two channels were used to acquire surface electromyography (sEMG) from the masseter (bilaterally), and the third channel was used to acquire the heart frequency (HF). The three signals were sampled at 800 Hz, with 8‐bit resolution	Orthodontic diagnosis was completed by the digital scanning of dental impression through dedicated soft‐ware (Clincheck Align Technology Inc. San Jose, California (USA)). This software allowed virtual simulations of orthodontic movements and guided the realisation of custom‐made clear aligners for each patient. All patients were provided with a precise sequence of clear aligners, to be replaced every 2 weeks, according to the standard protocol (28). Every month each patient was controlled in order to check compliance, orthodontic tooth movement and to perform routine inspections	The two groups of patients were followed for a period of 3 months and evaluated in three different moments (T0: screening, T1: evaluation after 1 month, T2: evaluation after 3 months) with instrumental (Bruxoff) recordings. The statistical analysis demonstrated that the number of SB episodes significantly reduced after 1 month follow‐up in the case group (*p* < 0.05, 95% CI). However, at T2 evaluation the number of SB episodes in the case group raised up to T0 levels with no statistical differences between T0 and T2 (*p* > 0.05, 95% CI). The number of total tonic contractions of the masseter's muscles in the case group reduced in a significant way after 1 and 3 months (*p* < 0.05, 95% CI), although the number of tonic contractions related to SB episodes in the case group, after a significant reduction in the first month (*p* < 0.05, 95% CI), raised up to T0 levels with no statistical differences between T0 and T2 (*p* > 0.05, 95% CI)	After the first month of clear aligners therapy, all patients in the case group showed a significant reduction in the number of SB episodes (*p* < 0.05). However, after 3 months SB values returned to baseline levels. The number of tonic contractions were reduced even after 3 months of orthodontic treatment (*p* < 0.05). Conclusions: While clear aligners seem to be capable to reduce clenching, i.e., occlusal load, in SB patients, the grinding activity seems to be not influenced by those appliances at least in the short term of the present investigation
Bargellini (2024) *Current Drug Delivery* Italy Cohort	65 participants screened; 51 included (21 M, 30 F, mean age 26.5 ± 3.5). Patients with SB received different oral appliances and were followed at 1 week, 1, 3, 6 and 12 months	17 patients treated with Invisalign clear aligners (mean age 18.9 ± 5.6), 11 with functional generating bite (mean age 53 ± 15.5), 7 with occlusal splint (mean age 44 ± 35.3), and 16 untreated controls (mean age 27.1 ± 1.4)	Instrumental diagnosis with portable EMG‐ECG holter (Bruxoff), requiring ≥ 2 episodes/h of sleep (mild to severe SB index)	Invisalign (Align Technology, San Jose, CA). Comparisons also included occlusal splint (resin) and functional generating bite (metal bite planes)	Occlusal splint reduced SB index after 1 week, 3, 6 and 12 months; FGB only after 12 months. Occlusal splints reduced phasic contractions for up to 6 months. Clear aligners did not affect SB index but reduced tonic contractions after 6 and 12 months	Oral appliances have distinct effects on SB and masticatory muscle activity: occlusal splints are most effective in reducing SB index; FGB shows late effect; clear aligners are neutral for SB index but reduce tonic contractions
Castroflorio (2018) Italy *Journal of Biological Regulators & Homeostatic Agents* RCT	60 subjects with sleep bruxism monitored over 6 months using EMG/ECG device	3 groups: Clear Aligner Therapy (CAT), Maxillary Occlusal Splint (MOS), Placebo Splint (PMS)	Portable EMG/ECG device (Bruxoff); SB index based on episodes/h	Full‐coverage clear aligners	CAT increased phasic contractions; MOS reduced masseter activity but increased tonic contractions; no significant change in SB index	CAT and MOS influence EMG patterns but do not significantly alter SB frequency; full‐coverage appliances recommended during orthodontic treatment
Colonna (2024) *Journal of Oral Rehabilitation* Italy RCT	The study design provided three different monitoring weeks with the smartphone application for AB evaluation, with a 1 week interval in between. The first week was a baseline monitoring during the week before starting the orthodontic treatment. The second and third monitoring weeks would provide patients to receive a passive aligner first, and then an active aligner	32 participants (12 males, 20 females; mean age 28.8 ± 3.9 years). Participants should be in good general health and the exclusion criteria were the presence of TMD pain	A smartphone—based application (BruxApp, World Medical Applications Srl, Italy) for a realtime report (i.e., EMA), which was specifically developed to report and monitor the frequency of AB behaviours	F22 clear aligners (Sweden & Martina, Due Carrare, PD, Italy)	The average reported frequency of the relaxed condition was 64.9%, 63.0% and 60.0% during the sessions without aligners, with passive aligners and with active aligners, respectively. ANOVA showed no significant differences in any of the AB behaviours within (i.e., between 7 days of evaluation) and between the monitoring sessions Considering the first session (i.e., without aligners), the average frequency of relaxed jaw muscles reports at the study population level was 64.9% (±23.2). Teeth contact (18.2% ± 15.9) and mandible bracing (12.0% ± 14.0) were the most frequently reported AB behaviours. During the second (i.e., with passive aligners) and third (i.e., with active aligners) sessions, the frequency of these conditions was as follows: relaxed jaw muscles 63.0% (±28.5) and 60.0% (±31.2), teeth contact 21.1% (±22.3) and 22.3% (±26.2) and mandible bracing 8.9% (±11.0) and 10.6% (±13.3)	The impact of our results in the orthodontic field is not negligible; clinicians can find support for the hypothesis that from a global point of view, wearing or not wearing aligners (passive and/or active) does not influence the frequency of AB behaviours at the short term
Heleiwa‐Ferioli (2024) *Journal of Clinical and Experimental Dentistry* Madrid Spain Quasi‐experimental	Evaluations were done prior to the start of treatment (T0), at 3 months after the start (T1), and at 6 months after the start	Data were collected from 100 patients: 58 females (58%) and 42 males (42%). There were 13 patients aged between 18 and 27 years (13%), 62 patients between 28 and 36 years (62%), and 25 patients between 37 and 45 years (27%)	Questionnaire about frequency of Bruxism Frequentist individual item reliability statistics	Invisalign transparent aligners	Before treatment with aligners, 64% of the patients had bruxism; 26 males (61%) had bruxism and 38 females (65.5%), with no statistically significant differences (*p* > 0.05). A significant association was found between the presence of bruxism and the age of the patients, being more frequent in those aged between 28 and 36 years (*p* < 0.05) Regarding the use of occlusal splints before starting treatment with aligners, 63% of patients did not use a splint compared to 37% who did. Concerning the item assessing the ‘feeling of clenching and/or grinding’, there was a general decrease in the sensation of clenching and/or grinding in most patients during the treatment follow‐up. This suggests that using Invisalign aligners might help reduce the feeling of tension or pressure in the oral area while wearing aligners	The frequency of bruxism does not depend on sex, but is related to age groups, the most affected being 28–36 years old patients. Statistically significant differences have been observed (*p* < 0.05) with notable reductions in: (1) Clenching and/or grinding sensation, (2) Sensation of contracted masticatory muscles, (3) Muscular pain of the masticatory muscles and (4) Pain of the temporomandibular joint. Additionally, (5) facial aesthetics and lip position experienced statistically significant differences (*p* < 0.05) without an increase or reduction being predominant. There is no relationship between the increase in headaches and the use of Invisalign transparent aligners, as no statistically significant differences were found (*p* < 0.05)
Liu (2017) *International Journal of Clinical and Experimental Medicine* Shandong, China RCT	All patients had received Invisalign treatment for at least 6 months In our study, tooth movement was not tested, allowing us to focus on the effect of the clear aligner appliance on the experimental results The Oral Behaviour Checklist (OBC) was completed at T0, i.e., before wearing the aligner; at T1, i.e., after 3 months of treatment; and at T2, i.e., after 6 months of treatment	23 female patients ranging from 20 to 28 years old (mean age 26.8 ± 2.4)	The Oral Behaviour Checklist (OBC), a DC/TMD (Diagnostic Criteria for Temporomandibular Disorders) axis II questionnaire	Invisalign Company (Invisalign, Align Technology, USA)	The OBC scores of T1 and T2 were significantly lower than that of T0 (*p* < 0.05), and no significant difference was revealed between T1 and T2. The OBC score significantly decreased initially and then changed with a minor increase from T1 to T2 (1) The OBC score significantly decreased betweenT0 and T1 (*p* < 0.05). (2) When measured at the MPP, the activity of the TA increased significantly between T0 and T1 (*p* < 0.05). During MVC, the activities of the TA and SCM at T1 were significantly higher than those of T0 (*p* < 0.05). At the MPP and MVC, we found no difference in TA, MM or SCM activities between T1 and T2	The orthodontic clear aligners have the relevant functional effect on the orofacial system, including oral parafunctional behaviours and the electromyographic activities of masticatory muscles. However, the effect of longer use on functional behaviours remains to be investigated and warrants further study
Manfredini (2018) *Progress in Orthodontics* Italy RCT	All subjects underwent an in‐home evaluation with a portable device (Bruxoff, OT Bioelettronica, Torino, Italy) that provides a simultaneous recording of EMG signals from both the masseter muscles as well as hearth frequency. The study design provided two consecutive recording nights, a night off, and then two additional recording nights with orthodontic retainers in situ	19 patients 14 females 28.3 (2.4) years old 25–35 age range	With a portable device (Bruxoff, OT Bioelettronica, Torino, Italy) that In this investigation, both the SB events (i.e., masseter contractions exceeding the 10% of maximum voluntary contraction [MVC] amplitude and preceded by a 20% increase in hearth rate) and the over threshold masseter contractions not preceded by the hearth rate increase were recorded. The latter were here called sMMA events. For each recording night, the number of SB episodes per sleep hour (SB index), the number of phasic, tonic, and mixed sMMA events per hour as well as the total sMMA events per night were calculated	For each individual, two orthodontic retainers, made of the same material that is commonly used to build invisible aligners, were manufactured. The retainers, one for the upper and one for the lower arch, were built on digital casts, as created after acquiring digital images of dental arches with an intraoral scanner (Trios 3 Shape, 3 Shape A/S, Copenhagen, Denmark). The same investigator with a 3‐year post‐graduate education in orthodontics (A.A.) manufactured the retainers for all participants by using a 1‐mm‐thick thermoplastic resin foil (Duran, Sheu Dental, Iserlhorn, Germany). The appliances were designed to cover all teeth up to the second molars. They were passively adapted to each tooth, to avoid any discomfort during the recording nights, and participants were asked to check for passive fit before starting any recordings	During the two nights without the retainers, five subjects had a SB index higher than the suggested threshold for PSG/SB diagnosis (i.e., > 4). Two additional subjects scored an average index higher than 4 during the nights with the retainers. Average SB index of the study population over the four recording nights was 3.3 ± 1.7 (range 0.2–7.9), with mean values of 3.0 ± 1.5 during the two nights without and 3.6 ± 1.9 with the retainers. As for sMMA, average number of events/nights was 73.4 ± 61.2. The mean number of phasic and tonic events/h was 7.4 ± 5.0 and 5.0 ± 1.4, respectively (Table 2). The distribution of contraction types was similar between the four recording nights. The average number of total sMMA events during the two nights without the retainers was 78.6 ± 65.6, and it was 67.9 ± 47.6 during the nights with the retainers. Between‐night differences were not significant for any of the outcome variables	Average SB index of the first two nights without the retainers was 3.0 ± 1.5, whilst the average values with the retainers in situ was 3.6 ± 1.9. ANOVA test showed the absence of significant differences between the four nights. Similarly, no differences were shown between the four nights as for the total number of sMMA events. Findings suggest the absence of relevant effects of invisible orthodontic retainers on sMMA in healthy individuals during the short‐term period
Pereira (2021) *Journal of Oral Rehabilitation* Brazil RCT	The sample comprised 38 Class I patients (mean age 22.08 years), divided by simple randomisation into two groups: OA group; orthodontic aligners (*n* 19) and FA group; fixed appliance (*n* 19). The frequency of AB was investigated by the ecological momentary assessment using an online device (mentimeter), during 7 following days at different timepoints, before and after appliance placement and in the 2nd, 3rd, 4th and 6th months of orthodontic treatment	38 Class I patients (mean age 22.08 years) 19 OA (8 female) 23.60 years old 19 FA (7 female) 20.56 years old	The frequency of AB was investigated by the ecological momentary assessment using an online device (mentimeter)	Group OA (*n* = 20) treated with Invisalign orthodontic aligners from Align Technology (Santa Clara, California, USA). The 3D planning was performed using the software ClinCheckTM Proversion 5,6 following the patients' needs and manufacturer's guidelines, and the aligners were changed at every 10 days Group (FA) was also composed of 20 patients treated with fixed appliances (slot 0.022 × 0.030″, 3 M Unitek, Monrovia, Calif). The appliances were placed up to the second molars following the same sequence of Nitinol wires (0.014″, 0.016″, 0.016 × 0.022″), respecting the individual needs of each patient. Orthodontic appointments were performed once a month by 2 orthodontic master program students at the post‐graduate clinics under supervision of an orthodontist with more than 15 years of experience	There was no difference between groups in the frequency of AB behaviours, with mean of 53.5% for group OA and 51.3% for FA. The most frequent behaviour was slightly touching the teeth, and in FA group, there was a significant reduction in this behaviour soon after appliance placement. The groups did not differ concerning the degree of anxiety, stress, catastrophizing, hypervigilance and facial pain	The orthodontic treatment performed with aligners or fixed appliances did not influence the frequency of AB during the 6 months of treatment
Pittar (2023) *Journal of Oral Rehabilitation* New Zealand and Italy Cohort	Participants were screened for oral parafunctional behaviours using the oral behavioural checklist Invitations to attend the screening appointment for phase two of the study were sent to 30 individuals with the lowest non‐functional OBC scores and 30 individuals with the highest non‐functional OBC scores, with an allocation ratio of 1:1. Recruitment was stratified and balanced by sex. At least 10 participants per group were required for the study to be sufficiently powered	The final sample of eligible selected participants consisted of 31 subjects: 16 in the low oral parafunction group (lower parafunction [LPF]; mean age 24.9 ± 5.2 years) and 15 in the high oral parafunction group (HPF; mean age 22.8 ± 4.0 years)	The OBC is a validated patient questionnaire that includes 21 items related to both nocturnal and diurnal oral parafunctional behaviours [20]. Each item has a total of five possible responses (weighted from ‘0’–‘4’), which relate to the frequency of certain oral behaviours	A 3D intraoral scan was taken using an iTero Element 5D (Align Technology) to facilitate PCA fabrication and assess occlusal contacts in centric occlusion. The ‘occlusal clearance tool’ was used to ensure at least 10 occlusal contacts were present in each participant. Dental models were printed using a NextDent 5100 AG 3D Printer out of NextDent for Ceramill Model 2.0 (NextDent B.V.) A single set of upper and lower PCAs were fabricated per participant out of 0.76 mm Zendura FLX (thermoplastic polyethylene‐polyurethane copolymer; Bay Materials LLC). The PCAs were shaped over participants' models via the pressure moulding method using a BIOSTAR VII pressure moulding device (Scheu‐Dental). Zendura FLX was chosen due to its previous use as a simulant material for clear aligners, its similar thickness and material properties to currently available aligners, and its relative affordability	The average length of the EMG recordings was 6.8 ± 1.3 h for the LPF group and 6.2 ± 1.3 h for the HPF group; these were not significantly different. While wearing the passive aligner, episode amplitude decreased significantly in both groups over time (*F* = 6.3; *p* = 0.003). The largest reduction was observed in the HPF group on Aligner Day 1, with further reductions seen after 8 days of continuous aligner wear in both groups. Despite this, there was no significant interaction of ‘group’ and ‘time’ (*F* = 2.2; *p* = 0.121) This indicates that the effect of wearing the aligners on mean episode amplitude did not differ significantly between HPF and LPF groups over time The number of contraction episodes per hour was not significantly influenced by group (*F* = 0.8; *p* = 0.386) or ‘time’ (*F* = 0.2; *p* = 0.976). There was no significant interaction of ‘group’ and ‘time’ suggesting that the aligner had no detectable effect on contractile episode counts in either group	This investigation aimed to determine the short‐term effect of PCAs on MMA, OD and TMD symptoms in adults with different levels of self‐reported oral parafunction. Analysis showed HPF individuals had significantly greater episode amplitude at baseline than their LPF counterparts. The introduction of the aligners was associated with a significant decrease in episode amplitude in all participants. No statistically significant differences were identified between the groups over time for episode frequency or duration, suggesting these aspects of muscle activity are not affected by the presence of PCAs or self‐reported oral parafunction
Saccomanno (2021) *Journal of Biological Regulators & Homeostatic Agents* Italy Cross‐sectional	After evaluation braces were prepared personalised based on previously taken impressions and cephalometric measures to correct the misalignment	236 consecutive patients (217 females and 19 males) Bracket type Ceramic Braces 27 (11.4%) Lingual Braces 1 (0.4%) Metal braces 75 (31.8%) Aligners 133 (56.4%)	Questionnaire by an online form service (Google Form service) asking: Do or did you suffer from teeth clenching? Do or did you suffer from bruxism? Before and during with the same intensity Before and during, but less before Before and during, but more before Before the orthodontic treatment During the orthodontic treatment Never	NR	All 236 patients responded to the online questionnaire, and we included 208 patients that used transparent or traditional braces. The only significant difference between the two groups was Bruxism The only statistically significative difference was related to bruxism, because we found a higher rate in patients treated with aligners than patients treated with metal braces	We found a higher rate of Bruxism in patients treated with aligners than patients treated with metal braces
Saccomanno (2022) *Journal of Biological Regulators & Homeostatic Agents* Italy Cross‐sectional	An anonymised questionnaire was diffused between patients who experienced clear aligner treatment	175 adult patients, over 18 years old	Questionnaire by an online form service (Google Form service) asking: Have you clenched or bruxed more during the orthodontic therapy? How much pain have you felt during the aligners therapy?	The most prevalent company of aligners was Invisalign (95.4%) and the 76% of patients had attachments	Half of patients had bruxism or clenching during the treatment (50.3%), while the 45.7% suffered of mucosal lesions. Moreover, only the 5.8% had a significant level of pain (over 8, in a scale between 1 and 10), the 24% experienced TMJ pain and the 26.9% had headache	In this study, half of patients had bruxism or clenching during the treatment (50.3%) but it isn't known the parafunction's level before the start of treatment

Abbreviations: NR, not reported; USA, United States of America.

Based on the outcomes reported, primary and secondary outcomes were defined a priori. Primary outcomes were those directly reflecting sleep or awake bruxism behaviour, including sleep bruxism index or episodes/h assessed by portable EMG devices, awake bruxism frequency assessed by ecological momentary assessment, validated self‐reported bruxism or oral behaviour questionnaire scores, and pain‐related outcomes such as temporomandibular or masticatory muscle pain. Secondary outcomes included instrumental or descriptive measures of masticatory muscle activity that do not directly define bruxism frequency, such as EMG amplitude, tonic or phasic contraction breakdown, episode duration, muscle recruitment patterns and subjective discomfort or muscle tension. This distinction was used to structure the narrative synthesis and to interpret findings according to their clinical relevance and diagnostic specificity. Hereafter are the main results sorted according to the distinct circadian manifestations.

### 3.5 Sleep Bruxism

Regarding primary outcomes, Bargellini et al. [21] observed a reduction in SB episodes after 1 month of aligner therapy; however, SB episode frequency returned to baseline after 3 months, indicating no sustained effect on the SB index. In a prospective cohort, Bargellini et al. [6] reported that occlusal splints significantly lowered the SB index over time, whereas aligners did not modify the index at any follow‐up point. Castroflorio et al. [10] and Manfredini et al. [23] found no significant changes in SB index associated with aligner or aligner‐like appliances. In contrast, several studies reported changes in secondary outcomes related to masticatory muscle activity. Bargellini et al. [21] and Bargellini et al. [6] observed reductions in tonic contractions at medium‐ and long‐term follow‐up, while Castroflorio et al. [10] reported increased phasic contractions with aligner use. Pittar et al. [24] found that passive clear aligners reduced the amplitude of sleep‐related contractions without affecting their frequency or duration, suggesting an effect on contraction intensity rather than on the occurrence of SB episodes.

### 3.6 Awake Bruxism

For primary outcomes, Colonna et al. [7] used ecological momentary assessment and reported no significant differences in awake bruxism (AB) frequency across baseline, passive and active aligner conditions. Similarly, Pereira et al. [19] found no differences in AB frequency between aligners and fixed appliances over 6 months, indicating a neutral effect of aligner therapy on AB behaviour.

Regarding secondary outcomes, Liu et al. [20] reported a reduction in Oral Behaviour Checklist scores after 3 months, which remained stable at 6 months, suggesting decreased self‐reported parafunctional behaviour. However, surface electromyography revealed increased short‐term activity in the temporalis and sternocleidomastoid muscles, indicating modifications in muscle recruitment patterns without corresponding changes in AB frequency.

### 3.7 Mixed/Self‐Reported (Not Specific to SB or AB)

Studies relying on self‐reported primary outcomes showed heterogeneous findings. Heleiwa‐Ferioli et al. [22] reported reductions in self‐reported clenching and grinding behaviours and temporomandibular joint pain after 3 and 6 months of aligner therapy, with no change in headache prevalence. Conversely, Saccomanno et al. [9], using a cross‐sectional questionnaire design, found a higher prevalence of self‐reported bruxism among aligner users compared with patients treated with metal brackets; however, the absence of pre‐treatment data and the potential for selection bias preclude causal interpretation, and this observation should be considered hypothesis‐generating. Similarly, Saccomanno et al. [8] reported that approximately half of the participants experienced clenching or bruxism during aligner therapy, with a subset also reporting temporomandibular pain and headache; nonetheless, because baseline prevalence was not assessed, these findings describe symptoms during treatment rather than increases attributable to aligner use.

### 3.8 Synthesis of Results

The data from the included papers were highly heterogeneous, making a meta‐analysis infeasible. So, a qualitative description was performed. The main methodological issue pertained to different manifestations of bruxism and methods to detect. A summary categorising each study as suggesting a neutral, protective or risk effect of aligner therapy on bruxism is presented in Table 2.

**TABLE 2:** Summary of included studies classified as neutral, protective, or risk in relation to aligner therapy and bruxism.

Author	Neutral	Protective	Risk
Sleep bruxism			
Bargellini (2017)	No persistent reduction in SB episodes, phasic contractions unchanged, return to baseline at 3 months	↓ tonic contractions at 1 and 3 months	
Bargellini (2024)	No effect on SB index across follow‐up	↓ tonic contractions at 6 and 12 months	
Castroflorio (2018)	No significant change in SB index across groups		↑ phasic contractions in aligner group; but do not significantly alter SB frequency
Manfredini (2018)	Passive retainers did not modify SB index or EMG activity in short‐term evaluation		
Pittar (2023)	Frequency and duration of contractions unchanged	↓ amplitude of contractions during sleep	
Awake bruxism			
Colonna (2024)	EMA showed no significant differences in AB frequency across baseline, passive and active aligner phases at the short term		
Pereira (2021)	There was no difference between groups in the frequency of AB behaviours at 6 months		
Liu (2017)	OBC stable after 3–6 months; EMG activity of temporalis and sternocleidomastoid increased	↓ OBC scores compared to baseline	
Mixed/self‐reported			
Heleiwa‐Ferioli (2024)	No improvement in headache	↓ self‐reported clenching/grinding, ↓ muscle contraction, ↓ TMJ pain (3 and 6 months)	
Saccomanno (2021)			Higher prevalence of self‐reported bruxism in aligner users compared with metal brackets
Saccomanno (2022)			~50% of patients reported clenching/bruxism during aligner therapy; some reported TMJ pain and headache; absence of baseline data limited conclusions

Abbreviations: AB, Awake bruxism; EMG, Electromyography; OBC, The Oral Behaviour Checklist; SB, Sleep bruxism; TMJ, Temporomandibular joint.

#### 3.8.1 Bruxism Diagnostic Framework

The interpretation of bruxism outcomes was informed by contemporary consensus definitions distinguishing possible, probable and definite SB or AB [1], based on history, clinical examination and instrumental assessment. However, this framework was not used as a formal eligibility criterion, as most included studies did not explicitly categorise participants according to these levels of diagnostic certainty. Instead, diagnostic approaches were recorded and considered descriptively. Instrumental assessments using portable electromyography, with or without electrocardiography, were interpreted as consistent with definite SB, while ecological momentary assessment via smartphone applications was considered indicative of definite AB. Studies relying on validated self‐reported questionnaires, such as the Oral Behaviour Checklist, were interpreted as assessing possible or probable bruxism, depending on the presence of clinical or instrumental corroboration. This diagnostic heterogeneity was accounted for during risk‐of‐bias assessment and contributed to the GRADE evaluation of evidence certainty, with greater weight given to studies using objective or real‐time assessment methods.

### 3.9 Confidence in Cumulative Evidence

The overall quality of evidence identified using GRADE's SoF tables was rated as very low for non‐randomised studies and moderate for randomised ones (Appendix S3), because studies presented a high risk of bias, which downgraded one point. For non‐randomised study designs (case–control, cross‐sectional, cohort and quasi‐experimental), the certainty of evidence was additionally downgraded by two points due to the inherent limitations of non‐randomised designs.

## 4 Discussion

### 4.1 Overview of the Evidence and Main Findings

The primary goal of this investigation was to systematically assess the relationship between clear aligner therapy (CAT) and bruxism, including SB and AB, evaluating whether these devices function as a neutral factor, protective agent or risk factor. Our findings reveal that this hypothesis cannot be definitively confirmed. The current literature on the effect of clear aligners on bruxism behaviours is notably limited and contradictory [19, 24], presenting contrasting views on whether aligners exacerbate or alleviate symptoms [23]. This systematic review therefore provides a pioneering, critical appraisal of existing evidence, underscoring the significant absence of a solid scientific consensus on the topic.

### 4.2 Findings Related to Sleep Bruxism

Evidence regarding the effect of clear aligners on SB presents a complex picture. EMG studies suggest that aligners may specifically reduce tonic contractions (clenching), while their effect on phasic activity (grinding) is less consistent [6]. A reduction in overall tonic contractions was observed in some studies at 6 and 12 months [6, 21]. However, many of these EMG effects are considered transient, often confined to the first month following appliance delivery, suggesting a functional adaptation of the orofacial system rather than a sustained therapeutic effect.

Instrumental findings concerning the overall SB index are even more inconsistent [6, 10]. Bargellini et al. [6, 21] reported sustained reductions in tonic contractions, while Castroflorio et al. [10], in a RCT, documented a significant, but temporary, increase in phasic contractions (tooth grinding) during the initial months of aligner therapy compared with placebo. Furthermore, studies evaluating passive devices, such as invisible orthodontic retainers, found no significant effect on the overall SB index [23]. Collectively, current evidence supports the notion that aligners should not be definitively classified as protective or harmful for SB, although they possess the capacity to modulate specific aspects of muscle activity (e.g., contraction intensity or type) [7].

### 4.3 Findings Related to Awake Bruxism

With respect to AB, the majority of objective and real‐time studies indicate a neutral effect of aligners [6, 7, 10, 19, 20, 23, 24]. Research by Colonna et al. [7] and Pereira et al. [19] found no significant difference in the frequency of AB behaviours (such as tooth contact, clenching, or grinding) when comparing aligners to fixed appliances or baseline conditions. A notable exception and nuance is the finding by Liu et al. [20], who reported a significant decrease in OBC scores after 3 and 6 months of aligner therapy compared with baseline. This suggests that while aligners may not consistently alter AB frequency, they may transiently modulate muscle activity or, importantly, patient self‐awareness of these behaviours. Although distinct patterns of muscle recruitment were observed in several studies, these objective findings did not consistently translate into clear, long‐term clinical significance.

### 4.4 Divergence in Subjective Reports and the Role of Individual Factors

Subjective symptom reports present the greatest complexity and underscore the high variability of individual responses. Heleiwa‐Ferioli and de la Cruz‐Vigo [22] reported significant subjective improvements in symptoms such as clenching/grinding perception, muscle pain and temporomandibular joint (TMJ) pain in aligner users. Conversely, Saccomanno et al. [9] found a higher prevalence of self‐reported bruxism among aligner users compared with fixed appliance patients. Subsequent studies reported that approximately half of patients (50.3%) perceived clenching or bruxism during aligner therapy, often co‐occurring with headache [8] or occlusal discomfort [24]. Occlusal discomfort was significantly greater in individuals with a history of high parafunction and somatization tendencies [24]. These divergences highlight that patient‐reported outcomes are critically dependent not only on the device itself but also on individual psychological and behavioural factors such as occlusal hypervigilance, anxiety and baseline somatization, which fundamentally influence both treatment expectations and clinical outcomes [24].

### 4.5 Clinical Implications and Suggested Adaptive Protocol

In cases where CAT is associated with increased pain or the perceived exacerbation of bruxism, particularly in patients identified with high occlusal hypervigilance [25, 26], a gradual adaptation protocol may be hypothetically indicated, in contrast to immediate full‐time wear (22–23 h/day). This approach, which has not been formally evaluated in the included literature, involves starting with short daily wear (e.g., 2–3 h) and incrementally increasing use over 1–2 weeks. The rationale is to allow the masticatory system to accommodate gradually, thereby reducing the likelihood of pain amplification, heightened muscle activity or persistent parafunctional behaviours.

Screening patients with validated questionnaires for hypervigilance, anxiety and catastrophizing [25, 26, 27] can help identify individuals who might benefit from this strategy. This suggestion is offered as expert clinical reasoning and should be interpreted as such, underscoring the crucial need for future studies to formally investigate the efficacy of adaptive wear protocols in susceptible populations. Individualised adaptation is paramount, emphasising the importance of monitoring patient comfort and behavioural responses alongside occlusal changes.

In the present review, the available evidence suggests that clear aligner therapy does not consistently increase objective indicators of bruxism activity, although transient changes in masticatory muscle symptoms and behaviours may occur. The prospective study by Tran et al. [28] reported that clear aligners were associated with mild tooth pain and mild masticatory muscle soreness of limited clinical relevance, without significant changes in pressure pain thresholds, suggesting no persistent sensitization of the trigeminal system. Importantly, muscle soreness was associated with oral behaviours and psychological factors such as anxiety and stress, indicating that behavioural adaptation rather than structural or pathological muscle changes may explain these findings. In parallel, ambulatory electromyographic investigations have shown that clear aligners may modify wake‐time masticatory muscle activity patterns, with some individuals exhibiting increased muscle activation or clenching episodes, possibly as an adaptive response to orthodontic forces or aligner presence [29]. However, these changes do not necessarily reflect clinically defined awake bruxism, as most studies did not apply standardised diagnostic criteria. Consistent with our findings, the currently available evidence indicates that aligners may influence short‐term muscle activity and subjective discomfort, but there is insufficient high‐quality evidence to conclude that clear aligner therapy induces or exacerbates sleep or awake bruxism as a defined clinical behaviour.

### 4.6 Interpretation of Qualitative Classification

The pragmatic classification of studies as neutral, protective, or risk served as a necessary qualitative tool to synthesise highly divergent evidence, but should not be mistaken for a definitive statement on the clinical role of CAT in bruxism. This categorisation helped to organise findings across various phenotypes, diagnostic methods and outcomes. A ‘protective’ designation (e.g., transient reductions in tonic contractions) does not imply sustained therapeutic benefit, nor does a ‘risk’ classification (e.g., increased self‐reported prevalence without objective data) necessarily indicate causality or clinical worsening. The frequent co‐existence of neutral and directional findings within the same investigation further complicates interpretation. This classification should be viewed as complementary to the narrative synthesis, supporting the conclusion that current evidence is insufficient for clear aligners to be conclusively defined as protective or harmful.

### 4.7 Methodological Limitations and Heterogeneity

A major limitation that undermines the possibility of meaningful quantitative synthesis is the marked methodological heterogeneity among the included studies. Considerable variation exists in diagnostic methods, encompassing portable EMG for SB and muscle activity, ecological momentary assessment (using mobile apps for AB, and self‐reported questionnaires (e.g., OBC) [6, 10, 19, 20]). Further variability was found in aligner type (active vs. passive/retainer), follow‐up periods (8 days to 12 months), and study populations. The profound heterogeneity in materials, appliance protocols and EMG acquisition/scoring criteria prevents meta‐analysis and reinforces the urgent need for standardised, well‐designed trials with larger samples and longer follow‐up. Diagnostic issues also must be outlined: few studies explicitly adopted the contemporary consensus framework differentiating possible, probable and definite SB or AB based on the combination of self‐report, clinical examination and instrumental assessment. Studies utilising portable EMGor EMG–ECG or ecological momentary assessment align more closely with definite bruxism, whereas questionnaire‐based studies reflect possible or probable bruxism. This implicit diagnostic uncertainty was a substantial contributor to methodological bias, highlighting the necessity for future studies to explicitly operationalise this framework to enhance comparability. Another limitation arises from study design. Findings from cross‐sectional and self‐reported studies must be interpreted cautiously. These designs cannot establish temporality, thus precluding differentiation between pre‐existing bruxism, treatment‐related perception and true modification of activity. In the absence of baseline assessment, symptoms reported during treatment are a descriptive observation, not evidence of a causal increase in prevalence attributable to aligner use. Self‐reported outcomes are inherently prone to bias from patient awareness, expectations and hypervigilance, further necessitating longitudinal designs with objective or real‐time diagnostic methods.

### 4.8 Strengths and Future Directions

Despite these profound limitations, this review provides significant strengths. It is the first systematic and critical synthesis of the available evidence on the aligner–bruxism interaction, conducted following rigorous PRISMA, PROSPERO and GRADE protocols. The analysis clearly identifies significant knowledge gaps and provides explicit directions for future research. Critically, future investigations must prioritise harmonised diagnostic criteria (adopting the possible–probable–definite framework), standardised instrumental methodologies (EMG protocols), and adequately powered RCTs with long‐term follow‐up. From a clinical standpoint, the findings strongly caution that, at present, scientific evidence is insufficient to regard aligners as either protective or harmful, reinforcing the necessity of individualised management and the urgent need for higher‐quality evidence.

## 5 Conclusion

Current literature on the relationship between clear aligner therapy and bruxism is characterised by substantial methodological heterogeneity, precluding a definitive meta‐analysis. While most data suggest an overall neutral effect, evidence remains contradictory, showing both transient reductions in tonic activity and sporadic increases in phasic grinding or subjective discomfort. Rather than identifying aligners as a definitive risk or protective factor, the available evidence highlights a critical lack of standardised diagnostic protocols. Consequently, no firm clinical conclusions can be drawn. Future research must prioritise harmonised diagnostic criteria and longitudinal designs to provide meaningful clinical guidance.

## Author Contributions

André Luís Porporatti worked on study conceptualization, design, data collection, data analysis, drafted the initial manuscript and approved the final manuscript as submitted. He was the first reviewer. Ângela Graciela Deliga Schroder worked on study conceptualization, design, data collection, data analysis and approved the final manuscript as submitted. She was the second reviewer. Aline Bastos de Barros worked on study conceptualization, design, data collection, data analysis and approved the final manuscript as submitted. Milena Sampaio Kuczera worked on study conceptualization, design, data collection, data analysis and approved the final manuscript as submitted. Yves Boucher worked on study conceptualization, design, data analysis, drafted the initial manuscript and approved the final manuscript as submitted.

## Funding

The authors have nothing to report.

## Ethics Statement

The authors have nothing to report.

## Consent

The authors have nothing to report.

## Conflicts of Interest

The authors declare no conflicts of interest.

## Supporting information


**Appendix S1:** Database search strategy.

**Appendix S2:** Articles excluded and the reasons for exclusion.

**Appendix S3:** ‘Grading of Recommendations Assessment, Development and Evaluation’ (GRADE) Summary of Findings (SoF) table.

## Data Availability

The data that supports the findings of this study are available in the Supporting Information of this article.
