# Supplementary material for: Clear Aligners and Bruxism: A Systematic Review
Source: J Oral Rehabil. 2026 Mar 17;53(6):1225–43. doi: 10.1111/joor.70189 (PMC13168834; doi:10.1111/joor.70189)
Supplement: Supplementary file 1 — Appendix S1: Database search strategy. [file JOOR-53-1225-s002.docx]

**Appendix 1** - Database search strategy (April 15^th^, 2025)

| **Database** | **Search** |
| --- | --- |
| **PubMed** | (bruxism[MeSH Terms] OR "sleep bruxism"[MeSH Terms] OR "sleep bruxism" OR "awake bruxism" OR bruxism OR parafunction OR parafunctions OR parafunctional OR attrition OR wear OR facets OR clench* OR gnash* OR grind*) AND ("aligner"[Title/Abstract] OR aligners[Title/Abstract] OR "clear aligner"[Title/Abstract] OR "clear aligners"[Title/Abstract] OR "Clear Orthodontic Aligners"[Title/Abstract] OR "esthetic aligner"[Title/Abstract] OR "esthetic aligners"[Title/Abstract] OR "transparent aligner"[Title/Abstract] OR "transparent aligners"[Title/Abstract] OR invisalign[Title/Abstract] OR "Soul Smile"[Title/Abstract] OR "Angel aligners"[Title/Abstract] OR Ezaligner[Title/Abstract] OR "Clear Correct"[Title/Abstract] OR Spark[Title/Abstract] OR SureSmile[Title/Abstract] OR Essix[Title/Abstract] OR Smartee[Title/Abstract]) |
| **Scopus** | TITLE-ABS ( "sleep bruxism" OR "awake bruxism" OR bruxism OR parafunction OR parafunctions OR parafunctional OR attrition OR wear OR facets OR clenching OR gnasinging OR grinding ) AND TITLE-ABS ( "aligner" OR aligners OR "clear aligner" OR "clear aligners" OR "Clear Orthodontic Aligners" OR "esthetic aligner" OR "esthetic aligners" OR "transparent aligner" OR "transparent aligners" OR invisalign OR "Soul Smile" OR "Angel aligners" ) |
| **Web of Science** | “sleep bruxism” OR “awake bruxism” OR bruxism OR parafunction OR parafunctions OR parafunctional OR attrition OR wear OR facets OR clenching OR gnasinging OR grinding (Topic) AND “aligner” OR aligners OR “clear aligner” OR “clear aligners” OR “Clear Orthodontic Aligners” OR “esthetic aligner” OR “esthetic aligners” OR “transparent aligner” OR “transparent aligners” OR invisalign OR “Soul Smile” OR “Angel aligners” OR “Clear Correct” (Topic) |
| **EMBASE** | ('sleep bruxism'/exp OR 'sleep bruxism' OR (('sleep'/exp OR sleep) AND ('bruxism'/exp OR bruxism)) OR 'awake bruxism'/exp OR 'awake bruxism' OR (('awake'/exp OR awake) AND ('bruxism'/exp OR bruxism)) OR 'bruxism'/exp OR bruxism OR parafunction OR parafunctions OR parafunctional OR 'attrition'/exp OR attrition OR 'wear'/exp OR wear OR facets OR clenching OR gnasing* OR 'grinding'/exp OR grinding) AND (aligner:ab,ti OR aligners:ab,ti OR 'clear aligner':ab,ti OR 'clear aligners':ab,ti OR 'clear orthodontic aligners':ab,ti OR 'esthetic aligner':ab,ti OR 'esthetic aligners':ab,ti OR 'transparent aligner':ab,ti OR 'transparent aligners':ab,ti OR invisalign:ab,ti OR 'soul smile':ab,ti OR 'angel aligners':ab,ti OR ezaligner:ab,ti OR 'clear correct':ab,ti OR spark:ab,ti OR suresmile:ab,ti OR essix:ab,ti OR smartee:ab,ti) |
| **Google Scholar** | bruxism AND aligners filetype:pdf |
| **OpenGrey** | Bruxism |
