# Supplementary material for: Clear Aligners and Bruxism: A Systematic Review
Source: J Oral Rehabil. 2026 Mar 17;53(6):1225–43. doi: 10.1111/joor.70189 (PMC13168834; doi:10.1111/joor.70189)
Supplement: Supplementary file 2 — Appendix S2: Articles excluded and the reasons for exclusion. [file JOOR-53-1225-s003.docx]

**Appendix Table 2 -** Articles excluded and the reasons for exclusion (n=3).

| **Author - year** | **Reasons for exclusion*** |
| --- | --- |
| 1. Gulzar 2024 | 1 |
| 1. Iwamoto 2021 | 1 |
| 1. Silvaroli 2023 | 4 |

Legend:

1. Studies with no use of aligners;
2. Studies in animals;
3. Literature reviews, abstracts from conferences, letters, case reports (<10 cases) and personal opinions;
4. full text not retrieved.
