# Supplementary material for: Clear Aligners and Bruxism: A Systematic Review
Source: J Oral Rehabil. 2026 Mar 17;53(6):1225–43. doi: 10.1111/joor.70189 (PMC13168834; doi:10.1111/joor.70189)
Supplement: Supplementary file 3 — Appendix S3: ‘Grading of Recommendations Assessment, Development and Evaluation’ (GRADE) Summary of Findings (SoF) table. [file JOOR-53-1225-s001.docx]

**Appendix 3:** Grading of Recommendations Assessment, Development and Evaluation" (GRADE) Summary of Findings (SoF) tables, using GRADEpro software:

| **Certainty assessment** | | | | | | | **Results** | **Certainty** | **Importance** |
| --- | --- | --- | --- | --- | --- | --- | --- | --- | --- |
| **№ of studies** | **Study design** | **Risk of bias** | **Inconsistency** | **Indirectness** | **Imprecision** | **Other considerations** |  |  |  |
| **Is there an association between clear aligner therapy and bruxism? We searched for studies based on a PECOS framework, within children and adults undergoing orthodontic treatment with clear aligners compared to fixed appliances, other appliances, or no treatment; Outcomes should include sleep bruxism or awake bruxism frequency, EMG indices, self-reported bruxism, pain, and related measures.**  **Participants (P) children and adults undergoing orthodontic treatment;**  **Exposure (E) the use of clear aligners;**  **Comparisons (C) fixed orthodontics or no comparison group;**  **Outcomes (O) presence or modification of SB or AB, as assessed by instrumental, clinical, or self-reported measures;** | | | | | | | | | |
| 6 | non-randomised studies  1 Case-Control  2 Cross Section  2 Cohort  1 Quasi- Experimental Studies | serious^a^ | not serious | not serious | not serious | not serious | Bargellini et al. (2017, case-control) showed a transient reduction in sleep bruxism episodes after one month of aligner therapy, returning to baseline by three months.  In a cohort follow-up, Bargellini et al. (2024) found that clear aligners did not change SB indices but reduced tonic contractions after six and twelve months.  Pittar et al. (2023) observed a short-term decrease in EMG episode amplitude without changes in contraction frequency, suggesting a mild neuromuscular adaptation.  Cross-sectional studies (Saccomanno et al., 2021; 2022) revealed that self-reported bruxism and muscle discomfort were frequent during aligner therapy, affecting about half of patients, though pain intensity was generally low and transient.  The quasi-experimental study by Heleiwa-Ferioli et al. (2024) reported a subjective decrease in clenching and masticatory muscle tension during Invisalign treatment, particularly in adults aged 28-36 years. | ⨁◯◯◯ Very low^b^ | IMPORTANT |
| 5 | Randomized studies  5 studies | serious^a^ | not serious | not serious | not serious | not serious | Most studies found that clear aligner therapy does not significantly alter bruxism frequency or electromyographic indices when compared to fixed appliances or placebo splints.  Pereira et al. (2021) reported no change in awake bruxism (AB) frequency during six months of treatment.  Colonna et al. (2024) confirmed that neither passive nor active aligners influenced AB behaviors in the short term.  Similarly, Manfredini et al. (2018) observed no significant difference in sleep bruxism (SB) indices or masseter activity with or without passive retainers made of aligner-like material.  Castroflorio et al. (2018) found that full-coverage aligners modified EMG contraction patterns, slightly increasing phasic and tonic episodes, but without changing SB frequency.  Only Liu et al. (2017) reported a reduction in oral behavior checklist scores after 3 and 6 months of aligner use, suggesting a temporary improvement in parafunctional awareness. | ⨁⨁⨁◯ Moderate | IMPORTANT |

#### Explanations

1. Using Critical Appraisal Tools, studies were classified as high risk of bias.
2. In the GRADE system, non-randomized studies are automatically downgraded by two points.
